# Health inequities in unscheduled healthcare for children with intellectual disabilities in Ireland: a study protocol

**DOI:** 10.12688/hrbopenres.12973.2

**Published:** 2020-07-16

**Authors:** Emma Nicholson, Edel Doherty, Suja Somanadhan, Suzanne Guerin, James Schreiber, Gerard Bury, Thilo Kroll, Meredith Raley, Eilish McAuliffe

**Affiliations:** 1 Centre for Interdisciplinary Research, Education and Innovation in Health Systems (IRIS), University College Dublin, Belfield, Dublin 4, Ireland; 2 J.E. Cairnes School of Business and Economics, National University of Ireland, Galway, University Road, Galway, Ireland; 3 UCD School of Nursing, Midwifery & Health Systems, University College Dublin, Belfield, Dublin 4, Ireland; 4 UCD Centre for Disability Studies, University College Dublin, Belfield, Dublin 4, Ireland; 5 School of Nursing, Duquesne University, Pittsburgh, PA, USA; 6 UCD School of Medicine, University College Dublin, Belfield, Dublin 4, Ireland; 7 Disability Federation of Ireland, Fumbally Court, Dublin 8, Ireland

**Keywords:** health inequities, intellectual disability, children, unscheduled healthcare

## Abstract

**Background:** Health inequities for children with intellectual disabilities  are prevalent within different health systems, and children with intellectual disabilites  have shorter life expectancies than the general population, higher mortality rates before the age of 17 and have a greater risk of potentially preventable hospitalisations. A health systems approach to research in this area provides a useful means through which research can inform policy and practice to ensure people with intellectual disabilities receive equitable healthcare; however, there is a paucity of evidence regarding how to address differences that have been described in the literature to date. The overall aim of this research is to establish the extent of health inequities for children with intellectual disabilities  in Ireland compared to children without intellectual disabilities with respect to their utilisation of primary care and rates of hospitalisation, and to gain a better understanding of what influences utilisation of primary care and emergency department services in this population.

**Methods and analysis: **The design of this research adopts a multi-methods approach: statistical analysis of health data to determine the extent of health inequities in relation to healthcare utilisation; discrete choice experiments to explore General Practitioners’ decision making and parental preferences for optimal care; and concept mapping to develop consensus between stakeholders on how to address current healthcare inequities.

**Discussion: **By applying a systems lens to the issue of health inequities for children with intellectual disabilities, the research hopes to gain a thorough understanding of the varying components that can contribute to the maintenance of such healthcare inequities. A key output from the research will be a set of feasible solutions and interventions that can address health inequities for this population.

## Introduction

The health needs of people with intellectual disabilities are often complex and this population are known to utilise health services more often than people without intellectual disabilities ; an effect that remains generally stable cross-culturally ^1, 2^. Children with intellectual disabilities have poorer reported health status than children without and such health disparities are more marked in children and young people compared to other age groups ^3^. This population has a shorter life expectancy than the general population, with higher mortality rates before the age of 17 in children with intellectual disabilities compared to those without ^4^. In Ireland, the mortality rate of people with intellectual disabilities under the age of 19 is seven times higher than the general population ^5^. Rates of hospitalisation also tend to be higher for this population ^3^ with findings from Australia showing that children with Down syndrome were hospitalised at a rate five times that of the general population ^6^ and, in Canada, they have higher ambulatory physician visits and a greater risk of hospitalisation due to injury, respiratory illness and diabetes ^4^. A greater risk of further ill-health is exacerbated by an increased risk of socioeconomic disadvantage experienced by children with intellectual disabilities ^7^.

Access to healthcare constitutes the fit between the individual and the health system ^8^ and is influenced by a myriad of complex factors such as availability, utilisation, effectiveness and equity ^9^. Access to quality primary care is associated with improved patient outcomes and reduced hospitalisation rates, however, universal access to primary care is not synonymous with equity of access ^10^. General practitioners (GPs) experience challenges while treating people with intellectual disabilities related to communication difficulties, deriving incomplete medical histories, lack of knowledge regarding existing supports, and a lack of training ^1, 11^. GPs have also expressed that time restrictions may impact upon the quality of care that they provide for patients with intellectual disabilities ^11^. Ambulatory care sensitive conditions are conditions which can be managed with access to timely and appropriate outpatient care ^12^. Better access to primary care may decrease utilisation of emergency departments and rates of hospitalisation for such conditions ^12, 13^.

There is a significant lack of information related to accessing healthcare services for children with intellectual disabilities in comparison to the general population in an Irish context. Ireland has a national database that records details pertaining to service provision for people with intellectual disabilities, which aims to elucidate the service needs of people with intellectual disabilities in Ireland. While it is a valuable resource, not all people with intellectual disabilities are registered with the database and needs pertaining to access and utilisation of primary care and hospitalisation rates are not recorded. Healthcare provision, planning and coordination tend to be poorer for people within the intellectual disabilities population compared to the wider population ^3^, and such inequities are amenable to change by improvement in quality of healthcare ^14^. The UN Convention on the Rights of People with Disabilities (UNCRPD) sets out that the guiding principles of the convention will need to be considered in relation to existing policy and practice. Article 25 of the convention states that health professionals are required to provide the same standard of care for people with disabilities and outlines the importance of specialist training and appropriate ethical standards in order to meet the needs of people with disabilities. Moreover, Article 31 recommends that state parties use statistical and research data to support policy planning that will give effect to the Convention and are obliged to identify and address barriers that affect the rights of people with disabilities. While it is important to establish any health disparities that exist for this population, it is also vital that models and strategies for reducing any existing inequalities are also addressed ^1^. Previous research has suggested that the quality of care GPs can provide people with intellectual disabilities is limited due to factors such as time and lack of knowledge ^11, 15^. However, to the best of our knowledge, no previous research has sought to systematically examine and model the trade-offs that influence GP referral practices when treating children with intellectual disabilities.

While international evidence suggests that people with intellectual disabilities, including children, experience inequality in accessing healthcare ^16^, there is a paucity of evidence relating to the decision-making by frontline staff and parents in relation to this population. Decision-making is critical within healthcare where limited resources are an ongoing concern and ultimately result in a complex interplay between stakeholder choices and behaviours, which often dictate where competing resources are allocated ^17, 18^. Recognising the complexity of the factors that lead to health disparities for this population, beyond establishing the differences in utilisation, will be critical to identifying avoidable determinants of health disparities and how these can be modified to improve healthcare provision for this population ^1^. For instance, continuity of care has been highlighted as a preference for parents of children with developmental disabilities ^4^, whereas socioeconomic disadvantages may influence access to care for this population ^8^. Moreover, risk factors for emergency department utilisation and hospitalisation may be unique for this population ^19^. Within health services research, public and patient preferences can inform policy and practice, as ensuring the consideration of all viewpoints will increase the likelihood of demands being adequately met when planning for service provision ^17^.

There are significant gaps in the evidence base around health inequalities for people with intellectual disabilities whereby, the evidence was generally of low quality and heavily skewed towards psychiatric interventions ^20^. A systems level approach to research would strengthen primary care and improve equitable service for people with intellectual disabilities ^15^. Much of the research makes use of health records and administrative datasets to describe healthcare utilization which can be highly informative and cost-effective ^21^, however, children with intellectual disabilities are frequently under-represented in such research due to misclassification and poor coding ^13, 22– 24^. National longitudinal data can provide appropriate evidence to address the limitations of administrative datasets for assessing healthcare utilisation ^22^. There is a small evidence base on rates of hospitalisation for physical health conditions for this population ^3^ but there is a paucity of evidence related to the decision-making that drives or inhibits this access. Emergency or unplanned admissions for children with intellectual disability create challenges for the provision of reasonable adjustments to support good quality care ^25^. In a review examining the factors that influenced access to secondary healthcare for people with intellectual disabilities and found that a myriad of issues affected a persons’ experience of care, including poor communication by staff, lack of skills and knowledge about working with people with intellectual disabilities, and poor signage and layout in hospitals ^26^.

The proposed research herein will provide information about access and utilisation of primary and emergency care of children with intellectual disabilities, particularly in relation to inequalities compared to children without an intellectual disability. Modelling the health status and service utilisation of children with intellectual disabilities at a population level is critical to determine their needs and priorities and to build a robust evidence base for policy, planning and service provision ^4^. Rigorous mixed-methods research, which adopts a systems approach, will support the identification of targeted strategies and interventions that will strengthen service provision through policy and practice ^15^. The current research project will aim to examine the utilisation of first-contact healthcare for children with intellectual disabilities in Ireland compared to children without intellectual disabilities, gain greater understanding of parental preferences and GP decision-making that drives this utilisation and finally, devise strategies for improved healthcare for this population during a time of large scale changes within the Irish health system

## Methods and analysis

**Figure 1.**
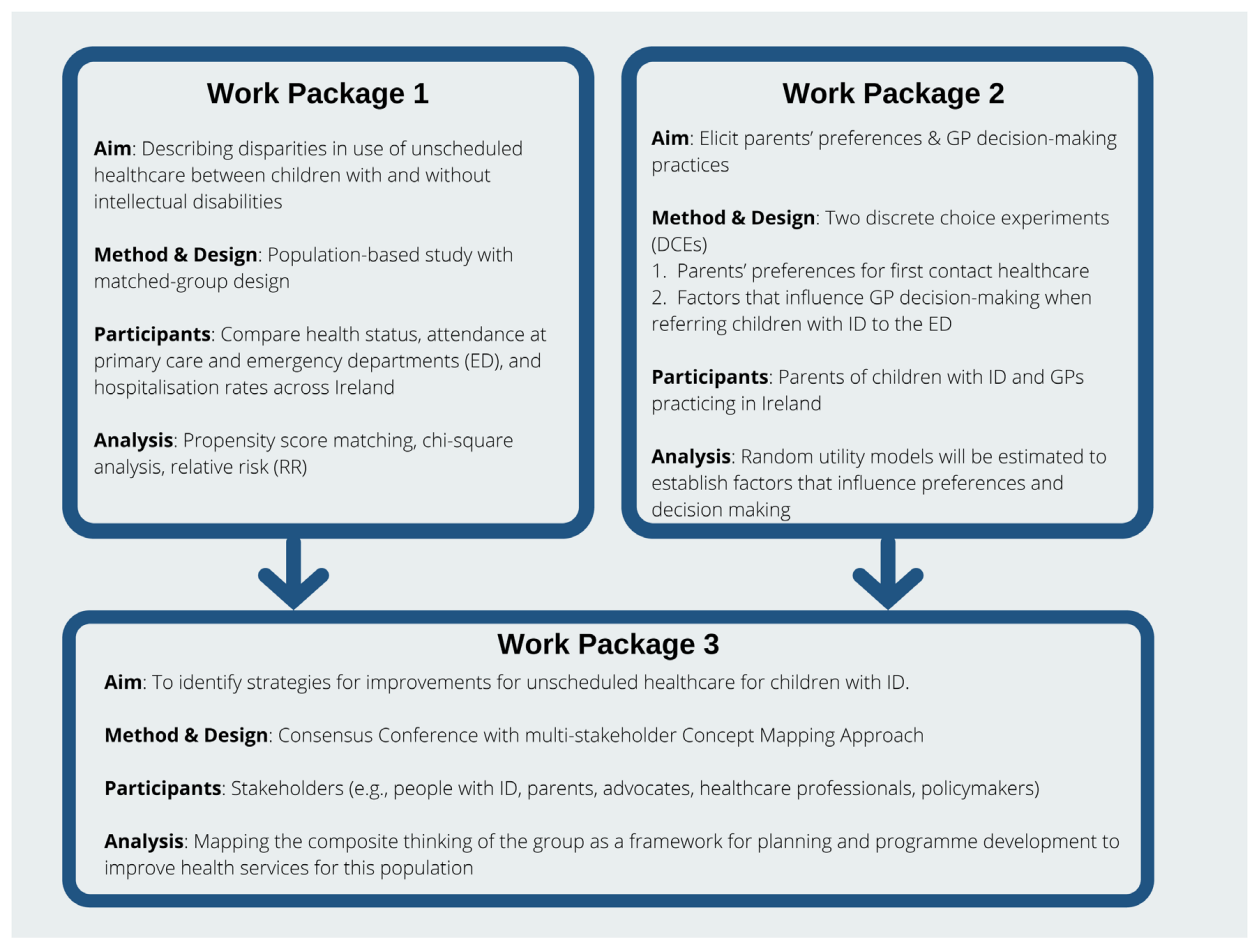
Outline of three work packages.

The proposed research will employ a multi-methods approach over three work packages to address the research objectives. Work package 1 will establish the extent of health inequities between children with and without intellectual disabilities with respect to access to health status, attendance at primary and emergency care, as well as rates of emergency hospitalisations. Work package 2 will examine the decision making and referral practices of GPs and elicit parental preferences for unscheduled healthcare for their children. Finally, work package 3 will seek to utilise the evidence that emerges from the first two work packages to develop strategies for improving access to unscheduled healthcare. The culmination of work packages 1 and 2 will seek to provide a comprehensive picture of the multifaceted issues that impact access and utilisation of healthcare for this population, while work package 3 aims to provide a model of strategies generated by stakeholders in order to foster meaningful impact of the research findings (see Figure 1).

### Work Package 1. Describing differences in healthcare utilisation and hospitalisation between children with and without intellectual disabilities

The aim of this work package will be to compare the use of unscheduled health services and hospitalisation of children under 16 years of age with intellectual disabilities to children under 16 years of age without intellectual disabilities. A cross-sectional population-based study with a matched-group design will be conducted and there will be two main data sources for this work package:

1. Existing datasets from an aligned study ^27^: These datasets will contain attendance data for paediatric populations from approximately 35 primary care practices and five emergency departments (approximately n = 367,405 pediatric patients) across Ireland, as well as in-patient data from children admitted to the hospital through the five emergency departments (approximately n = 96, 394 patients). The dataset documents attendances from 1 ^st^ of July 2013 to the 30th of June 2018 and children with intellectual disability will be identified using International Classification of Diseases 10 ^th^ edition (ICD-10) coding ^28, 29^, where possible. The prevalence of intellectual disability in Ireland is 1.4% of the entire population ^30^, however, it is expected that the prevalence of children with intellectual disabilities in these datasets will be lower than this figure due to the challenges of using administrative datasets for research purposes, as discussed above. Given these potential limitations, a second database will be used to ensure the research questions can be addressed if children with intellectual disabilities cannot be adequately identified using ICD-10 coding.
2. Growing Up in Ireland (GUI) study: GUI is a national longitudinal study of children and young people in Ireland which collects data from an Infant Cohort (n = 10, 000) and a Child Cohort (n = 8, 000). The survey collects data on health conditions, disabilities as well as attendance at and utilisation of health services including primary care, emergency departments and overnight stays in hospital ^31^. Prevalence of learning and intellectual disabilities in the child sample have been estimated at approximately 8%, however, it should be noted that the authors adopted a broad definition of intellectual disability ^32, 33^.

Children with intellectual disabilities will be matched by propensity score matching on specific variables (e.g., age, gender, medical card status, co-morbidities) with children without intellectual disabilities to allow for any differences between the two populations to be established. Given the greater number of males with intellectual disabilities in Ireland ^30^, it is likely that there will be more males in the intellectual disability group in both datasets.

The research questions for this work package are as follows:

1. Do children with intellectual disabilities have more attendances at primary care and the emergency department compared to those without in Ireland?
2. Do children with intellectual disabilities have a greater risk of emergency hospitalisations compared to those without ID?
3. What is the profile of children with intellectual disabilities who experience unscheduled hospitalisations in Ireland (i.e., age, gender, reasons for emergency hospitalisation [e.g., primary diagnosis], co-morbidities and length of stay)?
4. What is the relative risk of emergency hospitalisations for children with intellectual disabilities compared to those without?

***Data analysis plan:*** Propensity score matching will be used to match the children with intellectual disabilities to those without intellectual disabilities on specific covariates that may influence health outcomes. Imputation methods will be used where appropriate to address any missing data values. Descriptive statistics will be used to profile the patients, while *chi*-square tests and *t*-tests will allow comparisons to be made between the children with and without intellectual disabilities on the aforementioned factors and test the statistical significance of any variability. Relative risk (RR) will be used to determine if having an intellectual disability is a risk factor for emergency hospitalisations and 95% confidence intervals will be used to establish statistical significance.

### Work Package 2. Exploring and modelling the decision-making factors that influence referral practices when treating children with ID and eliciting parental preferences for care

A discrete choice experiment (DCE) is a survey-based methodology that elucidates the relative importance of certain factors or attributes that influence decision-making and preferences. They are increasingly being used in healthcare research as they provide real-world clinically-relevant scenarios to model decision-making at a more granular level by exploring the trade-offs that typically occur when multiple factors are considered during the decision making processes ^17, 34^. The underlying assumption of this methodology is that services, such as healthcare provision, can be broken into numerous characteristics, with individuals assigning differing values to each ^34^. For example, they have been used to examine preferences for access to primary care which highlighted that waiting time for an appointment was only important to patients when attending a new health concern and, from a parental perspective, if the appointment was for a child ^8^. DCEs have adequate external validity and have been shown to accurately mimic real-world decisions for choices within healthcare ^34, 35^. The proposed research will utilise DCEs in order to generate an understanding of the factors that influence GP decision making and referral practices when working with children with intellectual disabilities and to elicit parental preferences for unscheduled healthcare for their children:

1. **Exploring decision-making and referral practices of GPs when treating children with ID**. The attributes and factors that influence GP decision making and practices regarding referral to the emergency department and wider paediatric services will be examined using the DCE methodology.
2. **Eliciting parental preference for primary care for their children**. Using the above DCE methodology, the research will seek to model parents’ preferences regarding primary care for children with intellectual disabilities

In keeping with best practice in DCE design, the following four-step approach will be taken to conduct the DCEs:

**Step 1. Attribute development**: An exploratory step is crucial within a DCE design to establish the attributes that potentially influence decision making and preferences and to define the levels of each attribute ^36– 39^. Attributes may be patient, service or clinician-focused and can have numerous levels within them. For instance, in a DCE examining preferences for access to primary care, one key attribute was waiting time for an appointment with two levels being within 48 hours and in 4 days ^8^. An iterative approach is necessary to select attributes and levels that contribute to most variation in decision making ^38^. This will include a systematic review to identify the relevant literature in this area and qualitative inquiry ^39^ to explore factors that influence GP referral practices with this population and parent preferences related to their child’s healthcare.

The qualitative inquiry to develop the attributes will be an iterative two-step process consisting of conceptual development to establish the attributes and then refining the language used to ensure it is meaningful for the intended population ^38^. Interviews and focus groups ^38^ will be utilised with participants including both GPs and parents of children with ID. Purposive sampling will be used to ensure maximum variation of viewpoints are obtained. Topic guides for the qualitative data collection will be developed from the results of the systematic review. The qualitative data will be analysed using the constant comparison approach ^40^, which will allow for questions to be adapted in response to emerging data, which is particularly valuable within DCE ^38^.

**Step 2. Structured prioritisation exercise to finalise attributes**: Given the large number of possible attributes that may be relevant in the research, it is important to narrow the focus of the DCE to ensure that the included attributes and levels are feasible and meaningful to the wider research question and to safeguard the face validity of the DCE. A structured prioritisation exercise (SPE) will determine the relative importance of the attributes and factors that emerge from the qualitative work. These will be ranked in order of priority for inclusion in the DCE and to ascertain the levels required for each attribute. Considerations for attribute inclusion will comprise of issues relating to sample size calculation, ecological validity and ensuring adequate information is provided in the DCE. A panel made up of researchers, GPs, a health economist, parents and disability advocates will use the evidence from the SPE to decide on final design of the DCE.

**Step 3. Pilot study**: An experimental design will be used to generate the DCE choice cards that will allow for combinations of attributes and levels to be presented to participants in manageable subsets, presented in either table format or as vignettes. The result will be a series of hypothetical scenarios that each present combinations of attributes and levels to each participant. These will be piloted to test ease of use and to determine the length of time the DCE takes to complete, as well as establishing the plausibility to ensure clinical validity.

**Step 4. DCE and analysis**: Purposive sampling will be utilised when recruiting participants and sample size will be determined during the design of the DCE as it is contingent on the number of factors that emerge during the exploratory phase ^36^. Recruitment of GPs and parents will target participants to ensure representation based on factors that emerge during the research, e.g., to ensure a geographic spread amongst respondents. The DCE will record relevant participant information such as age, years of experience, training, socio-economic status etc.

Random utility models (including conditional logit models and mixed logit models) will be estimated to establish which factors affect decision making and preferences and the characteristics (e.g., age, level of experience, training, socioeconomic variables) of those making the decisions ^18^.

### Work package 3. A consensus conference to generate evidence-based strategies for improving access to healthcare for children with ID

The final work package will employ a multi-stakeholder concept mapping design within a consensus conference to identify strategies for improving access to healthcare for children with intellectual disabilities based on the results from the two previous work packages and as a means to lessen the gap between policy and practice. Evidence from work package 1 will establish the magnitude of health access disparities for children with intellectual disabilities, particularly relating to a risk of preventable hospitalisations, while the DCEs in work package 2 will explicate GP referral practices and parental preferences for unscheduled healthcare for their children. Work package 3 will build on these findings in order to address areas for improvement by discussing and generating strategies in partnership with patients, parents, healthcare professionals, disability advocates and policy makers. In order to identify workable strategies for improving access to healthcare, it is crucial to engage with stakeholders from all levels of the health system to ensure that the results are aligned with needs and priorities. Concept mapping ^41^ can provide a structured format for key stakeholders, with varying degrees of expertise, and support equal contribution to the development of strategies for improving access to care and discussing the feasibility of these strategies. This methodology has been successfully implemented to address complex issues in primary care where the delivery of primary care was comprehensively explored by stakeholders to provide a practice index for GP integration ^42^. Moreover, a modified version of this approach was used to generate priorities and strategies for improving access to maternity services among women with disabilities who have experienced domestic abuse ^43^.

Concept mapping is a methodology that seeks to map ideas or concepts generated by a diverse group of stakeholders through the integration of activities such as brainstorming and unstructured sorting with statistical analyses to map the ideas generated ^41^. The result is a visual map that represents the composite thinking of the group and can provide a framework for planning and programme development that incorporates complex elements perceived to be both important and feasible to stakeholders. A modified version of this approach will be adopted in this work package in the following steps:

1. **Sampling and recruitment.** Purposive sampling will be employed to ensure adequate representation across gender, disability-type and level of ID (i.e., mild/moderate to severe/profound). Up to 30 participants will be invited to participate and will include people with disabilities, parents of children with disabilities, disability advocates, policy makers, health and social care professionals (e.g., GPs, paediatricians, nursing staff, social care workers) and researchers and academics. Invitations will be sent out to GP surgeries, disability services, and children’s hospitals.

2. **Procedure**. The findings from work packages 1 and 2 will be presented to the group. Participants will then engage in a brainstorming session to derive strategies to address the issues, challenges and areas for improvement that emerge from the research in the previous work packages. Participants will then be asked to individually rank the strategies in terms of priority on a 5 point Likert scale ranging from 1 (Very High) to 5 (Not a priority) and in terms of feasibility on a 5 point Likert scale ranging from 1 (Very Feasible) to 5 (Impossible). This step is key in the production of data for concept mapping. The participants will then discuss the means through which the results of the can be used as a framework to enhance policy or practice in order to improve access to healthcare for children with ID.

3. **Analysis**. Findings will be synthesised and mapped to support the development of a set of recommendations for policy and practice to improve access to healthcare for children with ID. Hierarchical cluster-analysis will be used to rank and identify priority areas from the perspective of the different stakeholder groups. A bivariate plot of the two sets of ratings (priority and feasibly) will produce a ‘go zone’ graph to map the stated importance and feasibility of the strategies. The plot is divided into quadrants based on the average priority ( *x* axis) and feasibility ( *y* axis) scores where the top right quadrant will represent the strategies rates as being of highest priority and the most feasible. These ‘go-zones’ will identify the potential courses of action which are highly useful for planning purposes as they provide a detailed outline of the strategies that key stakeholders collectively view as important and feasible.

This procedure will enable the generation of a set of recommendations for policy and practice. Embedding stakeholder involvement throughout this process will provide a valuable mechanism to support meaningful and feasible impact of the research findings.

### Ethical considerations

The proposed research has been granted full ethical approval by the University College Dublin Research Ethics Committee (Reference: LS-19-64-Nicholson). All participants will provide written consent on their own behalf for their participation in the study.

### Public and patient involvement (PPI)

The project will adopt a disability-centred approach whereby patients, parents and people with intellectual disabilities will have input in the design, analysis and dissemination of the research throughout the project. At the outset of the project, a panel of PPI members comprising of parent and patient representatives from the disability community as well as disability advocates will be recruited to the project. This panel will contribute to the governance of the research and will be integral members of each work package sub-committee where they can advise and contribute to the design and analysis of the research. Members of this panel will also facilitate the consensus conference in work package 3.

## Study status

Work package 1: Early stages of analysis.

Work package 2: A systematic review to inform the qualitative inquiry was recently completed.

Work package 3: Not yet started.

## Discussion

The overarching aim of the research is to establish the extent of health inequities for children with intellectual disabilities in Ireland compared to children without intellectual disabilities, with a focus on their utilisation of primary care and rates of hospitalisation, to gain a better understanding of what influences utilisation and avoidable hospitalisations in this population. Applying a systems-approach, whereby the various components of the health systems in which the health inequities and the intricacies of the relationships between these will be considered, can provide evidence towards understating health inequities for this population and seek to identify interventions that can address them ^15, 44^. Drawing on existing evidence, while also being cognizant of the challenges that may arise, the proposed research will seek to build on previous work by drawing on multiple data sources to examine disparities in healthcare utilization while critically, identifying key areas for improvements in consultation with stakeholder groups.

While describing the health inequities experienced by people with intellectual disabilities is an important element for study, research must endeavour to establish the causal factors behind inequities, such as increased hospitalisations ^3^, and examine whether they can be considered avoidable and unjust ^1^ and critically, amenable to improvements in the quality of care ^14^. The inclusion of the DCE methodology can provide explanatory models that have important applications within policy making in health and, critically, can point to modifiable factors that contribute to any disparities in referral patterns experienced by this population. GPs are required to make highly complex decisions within health systems of scarce resourcing and thus, establishing the trade-offs they dictate, their behaviour is important for planning purposes ^37^. Moreover, preferences for healthcare beyond outcomes alone are important for planning quality care ^18^ and eliciting parents’ stated preferences for first contact care for children with intellectual disabilities will be key to identifying opportunities for interventions that can support meaningful change.

In keeping with the disability-centred approach of the project, ongoing and continuous knowledge exchange activities will be adopted to support an accessible and wide-reaching dissemination plan. Drawing on an evidence-based model for knowledge transfer of health research ^45^, dissemination will strategically target stakeholders with materials designed specifically for their needs and through avenues chosen to maximise their reach. Four key components will be considered when disseminating the research: messages, stakeholders, processes and contexts. For instance, workshops will be held with healthcare professionals to outline the results from the studies and receive feedback. A key output from the project will be a set of recommendations developed in collaboration with stakeholders that will be aimed at policymakers. Alongside traditional peer-reviewed publications, accessible materials, such as infographics, will be created to communicate the results from each work package to the disability community. These will outline the results in clear and accessible formats using lay terminology and will be designed with the PPI panel members and developed by a graphic designer to ensure their suitability for dissemination to the general population.

### Limitations

Potential inconsistencies in the reporting of intellectual disabilities within the Irish health system may be a limitation for the proposed research, as it may hinder our ability to accurately identify patients with ID from the data administrative systems. For instance, people with mild ID may be significantly underrepresented in the data systems as their care needs will not be considered as great as people with severe and profound intellectual disabilities. Understanding the extent of reporting of intellectual disabilities will be beneficial in and of itself, in order to raise awareness of the differences in health presentations for this population, which may not be widely known among those involved with practice and planning ^5^. Monitoring trends and identifying any health disparities for this population is crucial for the development of appropriate interventions that will facilitate good health for people with intellectual disabilities ^46^. The recent ratification of the UNCRPD requires the government to use research and statistical data to develop policies that give effect to the Convention ^19^ and the availability of appropriate data is key for policy and planning purposes.

## Conclusion

The proposed programme of research will apply a systems lens to the issue of health inequities for children with intellectual disabilities, specifically in relation to unscheduled healthcare. Across three work packages, the research will establish the extent of inequities for this population in relation to utilisation of unscheduled health services, elicit parents’ preferences for their children’s healthcare, as well as model the factors that influence GP decision-making. Finally, the study will seek to identify feasible solutions and interventions that can address health inequities for this population.

## Data availability

No data are associated with this article.
