# Supplementary material for: Health inequities in unscheduled healthcare for children with intellectual disabilities in Ireland: a study protocol
Source: HRB Open Res. 2020 Jul 16;3:3. Originally published 2020 Jan 23. [Version 2] doi: 10.12688/hrbopenres.12973.2 (PMC7431971; doi:10.12688/hrbopenres.12973.2)
Supplement: Peer review history

# Peer review: Reviewer response for version 2

**DOI:** 10.21956/hrbopenres.14203.r27672

**Published:** 2020-08-17

**Authors:** Lisa O'Leary

**Affiliations:** 1 Edinburgh Napier University, Edinburgh, UK

Thank for addressing all my previous recommendations, I have no further comments to make.

Is the study design appropriate for the research question?

Yes

Is the rationale for, and objectives of, the study clearly described?

Yes

Are sufficient details of the methods provided to allow replication by others?

Partly

Are the datasets clearly presented in a useable and accessible format?

Not applicable

Reviewer Expertise:

Intellectual disabilities, mixed methods, systematic reviews, complex interventions, mortality patterns in people with intellectual disabilities, respiratory health.

I confirm that I have read this submission and believe that I have an appropriate level of expertise to confirm that it is of an acceptable scientific standard.

# Peer review: Reviewer response for version 1

**DOI:** 10.21956/hrbopenres.14058.r27298

**Published:** 2020-05-07

**Authors:** Reema Harrison

**Affiliations:** 1 School of Public Health and Community Medicine, University of New South Wales, Sydney, Australia

This protocol presents the proposed study of the utilisation of primary and emergency care amongst children with intellectual disability to understand the influences on this population through a healthcare equity lens.

 The project addresses a significant and under-researched area that is of critical importance to health system equity, sustainability, value and quality. The program of work outlined integrates a range of relevant methods to generate the data required to address the proposed questions. The data quality relating to the identification of children with and without intellectual disability is unclear and given the lack of high-quality data identified elsewhere, it may be worth considering the implications of this for the proposed work.

 Areas for further consideration:

 It is notable that the terminology is a little inconsistent regarding intellectual disability, children and disability and would benefit from clarification. This may be in part due to reliance on literature relating to adult populations or disability more generally to form the development of the study but warrants review across the piece for clarity in some areas.

 Whilst a mixed-methods approach is identified, there is insufficient detail as to the process of data synthesis and how these studies collectively address the research questions. Currently, the work packages appear to flow one after the other but it is not clear as to how there will be a higher level synthesis of the knowledge. Overall, whilst the methods are well-described, there is insufficient detail in some of the areas to enable replication based on the protocol alone.

Is the study design appropriate for the research question?

Yes

Is the rationale for, and objectives of, the study clearly described?

Yes

Are sufficient details of the methods provided to allow replication by others?

Partly

Are the datasets clearly presented in a useable and accessible format?

Not applicable

Reviewer Expertise:

Health services research; healthcare quality; patient safety.

I confirm that I have read this submission and believe that I have an appropriate level of expertise to confirm that it is of an acceptable scientific standard.

# Response

**Published:** 2020-07-03

**Authors:** Emma Nicholson

**Affiliations:** University College Dublin, Ireland

This protocol presents the proposed study of the utilisation of primary and emergency care amongst children with intellectual disability to understand the influences on this population through a healthcare equity lens.

The project addresses a significant and under-researched area that is of critical importance to health system equity, sustainability, value and quality. The program of work outlined integrates a range of relevant methods to generate the data required to address the proposed questions. The data quality relating to the identification of children with and without intellectual disability is unclear and given the lack of high-quality data identified elsewhere, it may be worth considering the implications of this for the proposed work.

- The Introduction and Discussion have been amended to address potential international impact

**Areas for further consideration:**

It is notable that the terminology is a little inconsistent regarding intellectual disability, children and disability and would benefit from clarification. This may be in part due to reliance on literature relating to adult populations or disability more generally to form the development of the study but warrants review across the piece for clarity in some areas.

- This has been addressed and intellectual disability has been used throughout

Whilst a mixed-methods approach is identified, there is insufficient detail as to the process of data synthesis and how these studies collectively address the research questions. Currently, the work packages appear to flow one after the other but it is not clear as to how there will be a higher level synthesis of the knowledge. Overall, whilst the methods are well-described, there is insufficient detail in some of the areas to enable replication based on the protocol alone.

- Given there is no plan to specifically triangulate data, the term ‘multi-method’ has been used in place of mixed-methods. A figure has also been included to outline the project (see Figure 1). Greater detail has been provided in the method sections.

# Peer review: Reviewer response for version 1

**DOI:** 10.21956/hrbopenres.14058.r27143

**Published:** 2020-02-12

**Authors:** Lisa O'Leary

**Affiliations:** 1 Edinburgh Napier University, Edinburgh, UK

This is an important protocol for a potentially valuable study which has the potential to address health inequities experienced by children with ID in Ireland. I recommend this for indexing (subject to a few minor changes which I have specified in the summary below).

 A clear rationale has been provided for this study and the objectives of the study have been clearly described. The authors have clearly identified the evidence base for poorer health outcomes in children with ID compared to the general population (with specific reference to higher mortality rates and higher hospital admissions amongst children with ID compared to equivalent general population). They have made reference to studies undertaken both within an Irish and international context, in order to demonstrate this evidence.

 The authors have made reference to how inequitable access to health care may contribute to these poorer health outcomes in children with ID. They have also identified a gap in the evidence within an Irish context, wherein there is currently a lack of information related to accessing healthcare services for children with ID. They have referred to UNCRPD principles and gaps in research studies in order to emphasise the rationale for specifically investigating the complexity of factors that lead to health disparities in children with ID. The authors have emphasised the relevance of this within an Irish context (given the ratification of the UNCRPD). However, it would also have been helpful, if the authors could identify how the findings from this prospective study could have international impact on children with ID. This could increase the wider potential impact of the study. 

 The mixed methods three work package design appears appropriate for the research question **. **It would have been helpful if a figure was included in order to illustrate how each phase follows on and is integrated to the next, in terms of answering the research question. It would also be helpful to know what specific type of mixed method design is proposed? Do the authors propose to triangulate the results from each of the work packages (phases) of the study in any way?

 The authors have identified how they will establish the extent of health inequities between children with and without LD in relation to access to healthcare in the first phase of the study (work package 1). The authors have identified how they will match the cases. They have reported how they will represent the demographic profile of the participants. They have identified the intended statistical tests for drawing comparisons with the general population.

 However, I would recommend the following amendment for work package 1 phase, in order to ensure the data analysis plan in described in detail and allows replication by others. More information is required on the following in relation to work package 1. How is the proposed approach similar to the method used in Canada? Were there any adaptions that were made for the current study? How many children with ID are in this database? What specific age groups will the children be subdivided into for the purpose of analysis? What is the gender breakdown of the children in this database? How will missing data be treated? How many matched children are in this database?

 The authors have identified how they will use a discrete choice experiment to examine the decision making and referral process of GPs and identify parental preference for unscheduled care for treatment in phase 2 (work package 2). They have specified the four steps of this experiment. In the first attribute development step, they have identified that a systematic review and qualitative inquiry will be undertaken in order to identify the attributes that influence decision making. It would have been helpful if more detail was provided in relation to how the findings from the review will be explored further in the qualitative inquiry. They have specified how the second structured prioritisation exercise to finalise attributes will be undertaken. They have identified that the discrete choice experiment cards will subsequently be piloted in the third phase (specifically looking at length of time/ease of use of completion). It would have been helpful if some information about planned sample of participants in phase 3 could have been included (intended number of participants, age, gender breakdown etc.). The sampling, recruitment and analysis plan for step 4 is clearly identified.

 The authors have identified how the concept mapping approach in package 3 builds on findings from step 1 and 2. This is clearly reported and it is identified that the purpose of this is to elicit stakeholders’ recommendations for policy and practice. 

 Additional comments: the authors used the interchangeable terms of intellectual disability (ID) and disability throughout the transcript. It is suggested that if the focus of this study is just on children with an ID as opposed to children with any type of ‘disability’, then ID should be used instead of disability.

Is the study design appropriate for the research question?

Yes

Is the rationale for, and objectives of, the study clearly described?

Yes

Are sufficient details of the methods provided to allow replication by others?

Partly

Are the datasets clearly presented in a useable and accessible format?

Not applicable

Reviewer Expertise:

Intellectual disabilities, mixed methods, systematic reviews, complex interventions, mortality patterns in people with intellectual disabilities, respiratory health.

I confirm that I have read this submission and believe that I have an appropriate level of expertise to confirm that it is of an acceptable scientific standard, however I have significant reservations, as outlined above.

# Response

**Published:** 2020-07-03

**Authors:** Emma Nicholson

**Affiliations:** University College Dublin, Ireland

This is an important protocol for a potentially valuable study which has the potential to address health inequities experienced by children with ID in Ireland. I recommend this for indexing (subject to a few minor changes which I have specified in the summary below).

 A clear rationale has been provided for this study and the objectives of the study have been clearly described. The authors have clearly identified the evidence base for poorer health outcomes in children with ID compared to the general population (with specific reference to higher mortality rates and higher hospital admissions amongst children with ID compared to equivalent general population). They have made reference to studies undertaken both within an Irish and international context, in order to demonstrate this evidence.

The authors have made reference to how inequitable access to health care may contribute to these poorer health outcomes in children with ID. They have also identified a gap in the evidence within an Irish context, wherein there is currently a lack of information related to accessing healthcare services for children with ID. They have referred to UNCRPD principles and gaps in research studies in order to emphasise the rationale for specifically investigating the complexity of factors that lead to health disparities in children with ID. The authors have emphasised the relevance of this within an Irish context (given the ratification of the UNCRPD). However, it would also have been helpful, if the authors could identify how the findings from this prospective study could have international impact on children with ID. This could increase the wider potential impact of the study.

- The Introduction and Discussion have been amended to address potential international impact

The mixed methods three work package design appears appropriate for the research question. It would have been helpful if a figure was included in order to illustrate how each phase follows on and is integrated to the next, in terms of answering the research question.

- A figure has been included to illustrate the project (see Figure 1)

It would also be helpful to know what specific type of mixed method design is proposed? The term mixed methods reflects the variety of methods that will be used. Given there is no aim to triangulate

- Multi-method is a more appropriate term to describe the project and this has been amended throughout

 Do the authors propose to triangulate the results from each of the work packages (phases) of the study in any way?

- No, the data will not be triangulated

The authors have identified how they will establish the extent of health inequities between children with and without LD in relation to access to healthcare in the first phase of the study (work package 1). The authors have identified how they will match the cases. They have reported how they will represent the demographic profile of the participants. They have identified the intended statistical tests for drawing comparisons with the general population.

However, I would recommend the following amendment for work package 1 phase, in order to ensure the data analysis plan in described in detail and allows replication by others. More information is required on the following in relation to work package

1.         How is the proposed approach similar to the method used in Canada?

- The reference to this study has been removed

2.         Were there any adaptions that were made for the current study?

- See above

3.         How many children with ID are in this database?

- It is unclear at this stage how many children with ID are in the databases but it has

4.         What specific age groups will the children be subdivided into for the purpose of analysis?

- There is no plan to subdivide the children into age groups.

5.         What is the gender breakdown of the children in this database?

- Unknown at this stage, but it is expected that there will be more males than females in keeping with data from the CSO.

6.         How will missing data be treated?

- This has been addressed in the section ‘Data Analysis Plan’.

7.         How many matched children are in this database?

- Sample sizes have been provided where possible.

The authors have identified how they will use a discrete choice experiment to examine the decision making and referral process of GPs and identify parental preference for unscheduled care for treatment in phase 2 (work package 2). They have specified the four steps of this experiment. In the first attribute development step, they have identified that a systematic review and qualitative inquiry will be undertaken in order to identify the attributes that influence decision making. It would have been helpful if more detail was provided in relation to how the findings from the review will be explored further in the qualitative inquiry. They have specified how the second structured prioritisation exercise to finalise attributes will be undertaken. They have identified that the discrete choice experiment cards will subsequently be piloted in the third phase (specifically looking at length of time/ease of use of completion). It would have been helpful if some information about planned sample of participants in phase 3 could have been included (intended number of participants, age, gender breakdown etc.). The sampling, recruitment and analysis plan for step 4 is clearly identified. The authors have identified how the concept mapping approach in package 3 builds on findings from step 1 and 2. This is clearly reported and it is identified that the purpose of this is to elicit stakeholders’ recommendations for policy and practice.

Additional comments: the authors used the interchangeable terms of intellectual disability (ID) and disability throughout the transcript. It is suggested that if the focus of this study is just on children with an ID as opposed to children with any type of ‘disability’, then ID should be used instead of disability.

- The acronym ID has been removed and replaced with intellectual disabilities. Any reference to disability has also been removed and replaced with intellectual disabilities
